# Acute Effect of Bronchodilator on Intrathoracic Airway Wall Compliance in COPD Patients

**DOI:** 10.1007/s00408-022-00556-9

**Published:** 2022-07-18

**Authors:** Laura Pini, Giulia Claudia Ziletti, Manuela Ciarfaglia, Jordan Giordani, Claudio Tantucci

**Affiliations:** 1 Respiratory Medicine Unit, ASST - Spedali Civili di Brescia, Piazzale Spedali Civili 1, Brescia, Italy; 2 Department of Clinical and Experimental Sciences, University of Brescia, Brescia, Italy

**Keywords:** COPD, Airway wall compliance, Chronic bronchiolitis, Bronchodilator, Bronchial responsiveness

## Abstract

**Purpose:**

In patients with chronic obstructive pulmonary disease (COPD), bronchial responsiveness after acute administration of short acting bronchodilators is conventionally assessed by measuring the improvement of forced expiratory volume in the first second (FEV_1_) during a maximal forced expiratory maneuver. This study aimed to measure the variation of intrathoracic airway wall compliance (AWC) after acute administration of short acting beta-2 agonist in COPD patients since this might influence the final modification of airway caliber during maximal expiratory effort and the resulting bronchodilation as inferred by FEV_1_ changes.

**Methods:**

In a group of 10 patients suffering from COPD, intrathoracic AWC was measured at middle (50% of Forced Vital Capacity (FVC) and low (75% of FVC) lung volumes using the interrupter method during forced expiratory maneuver in basal conditions and after acute inhalation of albuterol (salbutamol) (400 mcg by MDI). Ten healthy subjects were examined similarly as a control group.

**Results:**

Lower values of baseline intrathoracic AWC at both lung volumes were found in COPD patients (1.72 ± 0.20 ml/cmH_2_O and 1.08 ± 0.20 ml/cmH_2_O, respectively) as compared to controls (2.28 ± 0.27 ml/cmH_2_O and 1.44 ± 0.22 ml/cmH_2_O, respectively) (*p* < 0.001). In COPD patients, AWC increased significantly at both lung volumes after salbutamol, amounting to 1.81 ± 0.38 ml/cmH_2_O and 1.31 ± 0.39 ml/cmH_2_O, respectively (*p* < 0.01), but the relative change was not different from that observed in controls.

**Conclusion:**

In COPD patients, AWC is reduced compared to controls, but after bronchodilator, the intrathoracic airways become more compliant. The consequent increased collapsibility under high positive pleural pressure could limit the airway caliber improvement seen after bronchodilator, as assessed by the FEV_1_ changes during the forced expiratory maneuver, underestimating the effective bronchodilation achieved in these patients.

## Introduction

During a forced expiratory vital capacity maneuver, the intrathoracic airway cross-sectional surface area upstream to the choke point, the airway wall stiffness and gas density with lung elastic recoil pressure are involved in determining effort-independent maximal expiratory flow at any given lung volume [1].

The intrathoracic airway distensibility and, in turn, their collapsibility in humans has been related to many interactive factors in vivo [2]. Among them, intrinsic elastic characteristics of the airway wall, namely the airway wall compliance (AWC), possibly linked to either the remodeling process or bronchomotor tone and the parenchyma-airways interaction, based on tethering forces acting through the alveolar attachments and lung elastance, have been thought to play an important role in determining the airway luminal area, mainly during high negative transmural airway pressure transients [3, 4].

In patients affected by chronic obstructive pulmonary disease (COPD) suffering from chronic bronchiolitis in whom lung elastic recoil and alveolar attachments are preserved, AWC appears to be the most relevant factor in determining intrathoracic airway distensibility and airway collapsibility during a forced expiratory maneuver at any given lung volume [5–9].

Therefore, if decreased, AWC might play a positive role in maximal expiratory flows and related measurements such as FEV_1_ at baseline by increasing airway wall stiffness with less airway collapsibility during forced expiration. Conversely, for the opposite reasons, if AWC increases after administration of a bronchodilator such as salbutamol, a beta-2 agonist with rapid onset of action conventionally used to assess the degree of bronchial responsiveness in these patients, that is known to reduce the airways smooth muscles tone [10, 11], this could negatively influence the post-bronchodilator improvement of the maximal expiratory flows and the related FEV_1_ variation.

This study aimed to measure AWC in clinically stable COPD patients suffering from prevalent small airway disease, namely chronic bronchiolitis, and to see the effect of acute bronchodilator administration on intrathoracic airway wall compliance.

## Methods

### Subjects

This study was performed at the Respiratory Medicine Unit of the University of Brescia—Spedali Civili Hospital of Brescia, Italy. Included in the study were 10 (7 male) consecutive COPD patients aged 55 ± 17 years with a diagnosis supported by the presence of risk factors, clinical assessment and objective measurements of lung function according to ATS/ERS criteria (FEV_1_/VC% < LLN) [12].

The COPD patients had to be clinically stable, never or ex-smokers, regularly treated with long-acting bronchodilators, with no functional evidence of relevant centrilobular pulmonary emphysema (as documented by a transfer factor coefficient greater than 70% of predicted) and without acute exacerbations in the previous 3 months. Exclusion factors were notable chronic comorbidities by self-reported medical history and medical records, if available, (heart failure, ischemic cardiac disease, chronic atrial fibrillation, overlap with obstructive sleep apnea, chronic respiratory failure, Parkinson’s disease and/or other neurological diseases, diabetes mellitus and/or other endocrine diseases) and the use of drugs such as beta-blockers and any other drugs capable of influencing bronchomotor tone. Ten (5 male) aged 43 ± 16 years never smoker healthy subjects were also enrolled as controls.

### Study Design

In the morning, at rest and while breathing room air, all subjects and each COPD patient, after an 8-h wash-out from short acting bronchodilators and 48-h wash-out from long-acting bronchodilators, performed spirometry (BIOMEDIN Instruments, Padua, Italy), wearing a nose clip and breathing through a flanged mouthpiece in sitting position. Slow vital capacity (SVC) and inspiratory capacity (IC) were measured twice using a bell spirometer at rest. Then, at least three acceptable and reproducible maximal full expiratory maneuvers were performed to measure forced vital capacity (FVC), maximal expiratory volume in the first second (FEV_1_), and maximal forced expiratory flows at different lung volumes.

Then, by single breath technique, alveolar volume (VA) and coefficient of transfer factor for CO (K_CO_) were measured twice only in COPD patients to obtain lung diffusing capacity for CO (DL_CO_), corrected for hemoglobin if needed (BIOMEDIN Instruments, Padua, Italy) for detecting the relative importance of the emphysematous component to their airflow obstruction, as stated above.

In each circumstance, the best values were retained for analysis. All tests were performed according to the ERS-ATS recommendations [12]. Predicted values of lung function parameters were those proposed by the European Community for Coal and Steel [13].

Subsequently, another three acceptable and reproducible maximal full expiratory maneuvers were performed 20–30 min. after inhalation (MDI plus spacer) of salbutamol (400 mcg), a short acting beta-2 agonist. Either in baseline condition or after bronchodilator administration, AWC was measured both in patients and controls at lung volumes corresponding to 50% and 75% of the expired volume starting from total lung capacity during baseline forced vital capacity maneuver. At middle lung volume, AWC is mainly related to the compliance of the downstream segment of larger intrathoracic airways, while at low lung volume, AWC reflects the compliance of the downstream segment, including also smaller intrathoracic airways. To avoid the effects of extra-thoracic airways on the Pao change dynamics as much as possible, the subjects and patients were asked to perform these maneuvers by placing their hands on the cheeks to prevent bulging.

The system built to obtain AWC consisted of a pneumotachograph (Hans Rudolph, model 4700, H. R., Kansas City) connected to a pressure differential transducer (MP 45 ± 2 cmH_2_O, Valydine Northridge) to measure flow and volume. The mouth pressure was measured at lateral pressure tap located between the subject and rapid interrupter valve by a rigid polyethylene catheter (1.7 mm id) connected to a pressure differential transducer (MP 150 ± 15 cmH_2_O, Valydine Northridge). The airflow interruption was performed using a computer-driven rapid interrupter valve (model R/-003, series L#004, Aeromech Devices Ltd. Almonte, Ontario, Canada), activated when 50% and 75% of the lung volume were expired from total lung capacity during a forced vital capacity maneuver, corresponding to MEF_50_ and MEF_75_, for a closure-time of 100 ms. The times for rapid interrupter valve activation were previously obtained from baseline maximal expiratory volume/time curve and given to a computer that started to compute the prefixed intervals to airway occlusions when an expiratory flow threshold of 20 ml/s was detected. The flow, volume and pressure signals were amplified, then filtered and digitized at a 500 Hz frequency (Ac Bridge Amplifer abc Module Raytech Instruments) and displayed on the computer monitor in real-time.

**Fig. 1:**
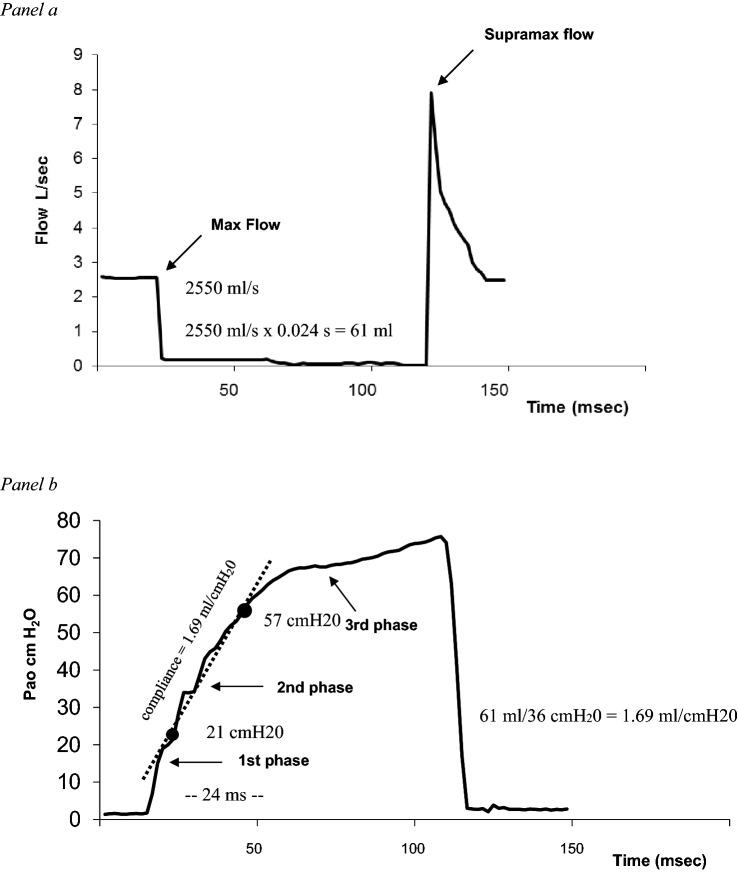
These graphs show the method used to measure AWC in a representative COPD patient at 50% of FVC during a maximal expiratory maneuver. In *panel * (**a**), the time-course flow dynamic is depicted during abrupt airflow interruption lasting 100 ms. Note the supramaximal expiratory flow occurring soon after the reopening of the rapid interrupter valve. In *panel *, **b** the corresponding relationship between Pao and time (volume) is shown with the procedure for determining AWC by linearly fitting the second phase of Pao change

To compute AWC, we used the method proposed by Ohya and coworkers that estimated from the mouth pressure–time curve the intrinsic elastic properties of the airway wall of the segment downstream the choke point obtained after abrupt airflow interruption during forced expiration [14]. Briefly, the change of gas volume flowing into the downstream airways segment after flow interruption at the mouth can be obtained by multiplying the interruption time with the maximal flow immediately before interruption because the maximal flow is maintained for a short period after airflow interruption. The mouth pressure change (Pao) was used for the pressure change of the airways downstream segment. Since pressure changes in downstream segment due to gas flowing downstream can be observed as changes in Pao, the Pao-time curve can be converted to a pressure–volume curve for downstream airway segment. Thus, downstream of the choke point, AWC can be obtained by linearly fitting the slope of the initial part of the second phase of the Pao-time relationship (Fig. 1). In fact, while the first phase of the abrupt rise of Pao represents the pressure increase by the instantaneous interruption of the flow itself, the second phase reflects the almost linear pressure change inside the airways downstream the choke point as a function of the volume that accumulates only in the downstream segment of airways and easily computed by the product of interruption time and maximal flow before the interruption. The curvilinear third phase of the Pao change approaches the alveolar pressure value (PA) that remains constant during shuttering since the lung volume (much larger) shows little variations even after the alveolar air (much smaller) has flowed into the collapsed segment. Also, the pleural pressure does not vary during this procedure, making the changes of transmural airway pressure downstream to the choke point equal to those shown by the Pao.

The study was performed in accordance with the Helsinki declaration and was approved by the University Ethics Committee at the Department of Clinical and Experimental Sciences (number 18/2018). All participants signed written informed consent upon enrolling.

### Statistical Analysis

The results are shown as mean ± standard deviation if not otherwise specified. Comparisons within groups were performed using a Wilcoxson signed-rank test for paired data. Comparisons between groups were performed using a Wilcoxson rank-sum test for unpaired data. Correlations between variables were performed using Pearson’s linear regression, and the coefficients of determination were computed.

Differences with p less than 0.05 were considered significant. Statistical analysis was made using the software GraphPad Prism Version 4.0.

## Results

The anthropometric characteristics of COPD patients and healthy subjects are shown in Table 1. The COPD patients suffered from mild-to-moderate airflow obstruction. Although they were older than controls, the age difference was not statistically significant.

**Table 1:** Anthropometric characteristics of healthy subjects (Controls) and COPD patients

	Controls	COPD	p
Age (yrs)	46.3 ± 16.4	55.4 ± 16.9	n.s
Sex (M/F)	5/5	7/3	n.s
Weight (Kg)	72.5 ± 12.7	75.7 ± 18.3	n.s
Height (m)	1.67 ± 0.12	1.68 ± 0.10	n.s
BMI (Kg/m^2^)	25.9 ± 3.9	26.8 ± 5.9	n.s

Data are mean ± SD

Baseline respiratory functional parameters and their changes after bronchodilator of both healthy controls and COPD patients are reported in Table 2 and Table 3, respectively. While no significant differences were observed after acute salbutamol inhalation in controls, many parameters were improved in COPD patients. Although even FEV_1_ and FVC significantly increased, their average improvement was less than 10% predicted and less than 12% from baseline and 200 ml, showing no bronchial responsiveness in this group of COPD patients.

**Table 2:** Respiratory functional parameters in basal condition and after salbutamol in healthy subjects

	Baseline	Post-bronchodilator	*p*
VC (l)	4.48 ± 1.11	4.46 ± 1.04	n.s
VC (% pred.)	118.2 ± 14.7	118.1 ± 15.1	n.s
IC (l)	3.13 ± 0.68	3.21 ± 0.73	n.s
FVC (l)	4.42 ± 1.10	4.34 ± 1.07	n.s
FVC (% pred.)	120.9 ± 16.1	118.7 ± 15.6	n.s
FEV_1_ (l)	3.68 ± 0.93	3.70 ± 0.96	n.s
FEV_1_ (% pred.)	121.2 ± 18.7	121.4 ± 16.8	n.s
FEV_1_/VC (%)	82.11 ± 5.17	82.61 ± 4.31	n.s
FEV_1_/VC (% pred.)	103.3 ± 5.6	104.0 ± 4.4	n.s
PEF (l/s)	8.66 ± 2.54	8.86 ± 2.54	n.s
PEF (% pred.)	113.4 ± 18.9	116.1 ± 19.2	n.s
MEF_25%_ (l/s)	7.98 ± 2.31	8.16 ± 2.35	n.s
MEF_50%_ (l/s)	4.92 ± 1.20	5.25 ± 1.44	n.s
MEF_75%_ (l/s)	1.69 ± 0.72	1.75 ± 0.83	n.s
FEF_25-75%_ (l/s)	4.02 ± 1.07	4.19 ± 1.29	n.s
FEF_25-75%_ (% pred.)	108.9 ± 22.7	112.0 ± 23.4	n.s

Data are mean ± SD

**Table 3:** Respiratory functional parameters in basal condition and after salbutamol in COPD patients

	Baseline	Post-bronchodilator	*p*
VC (l)	3.92 ± 1.00	4.11 ± 0.93	n.s
VC (% pred.)	104.7 ± 14.6	108.8 ± 12.4	< 0.02
IC (l)	2.70 ± 0.60	2.91 ± 0.58	< 0.01
FVC (l)	3.67 ± 1.10	3.89 ± 1.01	< 0.02
FVC (% pred.)	101.0 ± 16.5	107.0 ± 12.2	< 0.01
FEV_1_ (l)	2.21 ± 0.91	2.42 ± 1.00	< 0.02
FEV_1_ (% pred.)	73.6 ± 17.2	80.2 ± 15.2	< 0.02
FEV_1_/VC (%)	54.9 ± 10.2	57.9 ± 10.7	n.s
FEV_1_/VC (% pred.)	70.6 ± 10.8	74.4 ± 11.1	< 0.05
PEF (l/s)	6.10 ± 1.85	6.53 ± 1.65	< 0.01
PEF (% pred.)	80.8 ± 18.0	86.3 ± 13.7	< 0.01
MEF_25%_ (l/s)	3.76 ± 2.00	4.02 ± 2.15	n.s
MEF_50%_ (l/s)	1.47 ± 0.92	1.83 ± 1.53	n.s
MEF_75%_ (l/s)	0.41 ± 0.33	0.54 ± 0.56	n.s
FEF_25–75%_ (l/s)	1.10 ± 0.79	1.37 ± 1.24	n.s
FEF _25–75%_ (% pred.)	29.7 ± 13.7	36.1 ± 22.0	n.s
DLCO [mmol.min^−1^ kPa^−1^]	5.32 ± 1.0		
DLCO (% pred.)	67.7 ± 11.6		
KCO [mmol.min^−1^ kPa^−1^ L^−1^]	1.09 ± 0.24		
KCO (% pred.)	71.4 ± 10.3		

Data are mean ± SD

Both DL_CO_ and K_CO_ were slightly reduced in these COPD patients suggesting small airways disease with no or only mild centrilobular emphysema as the prevalent mechanism of their chronic airflow obstruction (Table 3).

**Fig. 2:**
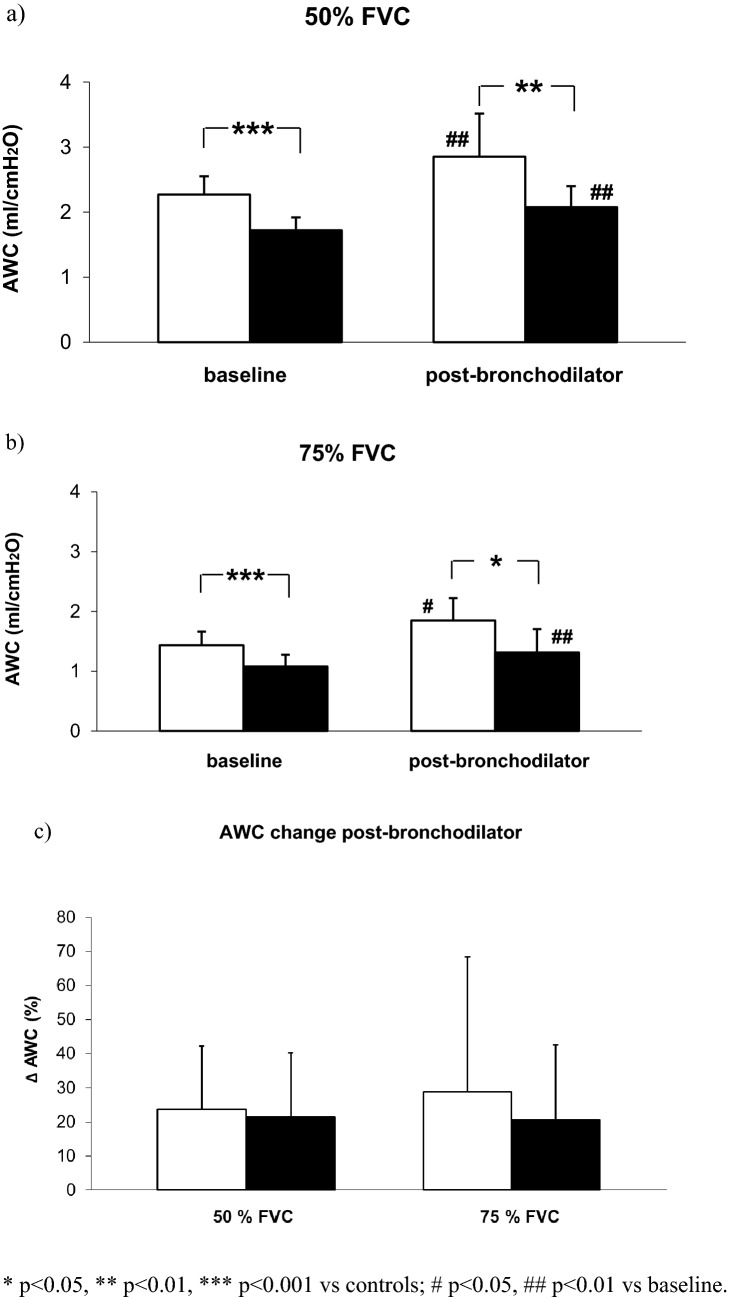
The average values of AWC in healthy subjects (white columns) and COPD patients (black columns) measured at 50% and 75% of FVC in baseline condition and after inhalation of salbutamol (400 mcg by MDI plus spacer) and the relative percent changes after bronchodilator.

Baseline AWC either at 50% or 75% of FVC was significantly higher in controls than in COPD patients amounting to 2.28 ± 0.27 ml/cmH_2_O vs. 1.72 ± 0.20 ml/cmH_2_O (*p* < 0.001) and 1.44 ± 0.22 ml/cmH_2_O vs 1.08 ± 0.20 ml/cmH_2_O (*p* < 0.001), respectively. Also after bronchodilator, either at 50% or 75% of FVC, AWC was significantly higher in controls than in COPD patients, amounting to 2.84 ± 0.63 ml/cmH_2_O vs 2.08 ± 0.32 ml/cmH_2_O (*p* < 0.01), and 1.81 ± 0.38 ml/cmH_2_O vs 1.31 ± 0.39 ml/cmH_2_O (*p* < 0.05), respectively (Fig. 2a and Fig. 2b).

In both groups, ACW significantly increased after salbutamol (*p* < 0.01 for COPD and *p* < 0.05 for controls), either at 50% or 75% of FVC (see Fig. 2a and Fig. 2b), with no significant difference between the two groups (Table 4 and Fig. 2c).

**Table 4:** Airway wall compliance (AWC) in basal condition and after salbutamol in healthy subjects and COPD patients at 50% and 75% of FVC with relative percent changes

Controls	Baseline	Post-bronchodilator	*p*
AWC FVC 50% (ml/cmH_2_O)	2.28 ± 0.27	2.84 ± 0.63	< 0.01
AWC FVC 75% (ml/cmH_2_O)	1.44 ± 0.22	1.81 ± 0.38	< 0.05
Δ AWC FVC 50% (%)		23.7 ± 18.5	
Δ AWC FVC 75% (%)		28.9 ± 39.5	
COPD	Baseline	Post-bronchodilator	
AWC FVC 50% (ml/cmH_2_O)	1.72 ± 0.20	2.08 ± 0.32	< 0.01
AWC FVC 75% (ml/cmH_2_O)	1.08 ± 0.20	1.31 ± 0.39	< 0.01
Δ AWC FVC 50% (%)		21.4 ± 18.7	
Δ AWC FVC 75% (%)		20.6 ± 21.8	

Data are mean ± SD. *P* values are between baseline and post-bronchodilator measurements

*p < 0.05, **p < 0.01, ***p < 0.001 vs controls; #*p* < 0.05, ##*p* < 0.01 vs baseline

No correlations were found between FEV_1_ (either as % pred. or % baseline) and ACW at 50% and 75% (either as absolute changes or % baseline) in COPD patients. In controls who showed no significant mean FEV_1_ changes after bronchodilator, 60% had FEV_1_ post-salbutamol unchanged (*n* = 2) or reduced (*n* = 4) compared to baseline.

## Discussion

The study results suggest that AWC is lower in COPD patients with mild-to-moderate airflow obstruction than in healthy controls both at middle lung volume involving large intrathoracic airways and at low lung volume, likely involving both large and small intrathoracic airways. The unremitting remodeling process affecting the structure of the central and mostly peripheral airways wall in COPD patients with chronic bronchiolitis may explain these functional features [15, 16]. However, these findings were opposite to what has been shown in other studies where AWC was found normal or increased in COPD patients compared with healthy control subjects [17, 18]. We have no clear explanation for these conflicting results. Heterogeneity of AWC measurements between COPD patients, according to the prevalent underlying disease of airflow obstruction (i.e., emphysematous vs bronchiolitic COPD patients) and small sample size could play a role, as well as the site of AWC measuring, central vs peripheral airways.

Looking at histopathological changes described in central and peripheral airways of COPD patients with chronic bronchiolitis and no or mild centrilobular emphysema typically showing an extensive sub-epithelial and particularly peri-adventitial fibrosis, it is hard to believe AWC can be increased, causing a greater distensibility of their bronchial and bronchiolar wall. In contrast, the chronic remodeling process that damages the structure of the central and mostly peripheral airways in COPD patients leading to progressive thickening and scarring of the bronchial and bronchiolar walls, may better fit with our data and explain these findings.

Conversely, a lower AWC conferring a greater airway stiffness may allow higher maximal expiratory flows for the same degree of airway obstruction according to the tube wave-speed theory described in the following equation (V,max = A × [A/ρ × Ptm/A]^0.5^), where V,max = maximal flow rate, A = cross-sectional surface area, Ptm = transmural pressure, ρ = gas density [19]. In addition, a lower AWC may offer an increased elastic load to the airway smooth muscle to limit an excessive reduction of airways lumen during bronchoconstriction [20].

On the other hand, using different approaches, several studies have shown a reduced airway distensibility in asthma, likely due to the modifications of intrinsic mechanical properties of the airway wall following the remodeling process occurring in both central and peripheral airways, where lung elastic recoil and alveolar attachments are usually preserved, as is the case in COPD patients with chronic bronchiolitis [2, 21–27]. Although obtained in asthmatic patients with airway wall remodeling, these findings indirectly support our results.

In both our groups, baseline AWC was less at lower lung volumes during forced maximal expiration when the downstream segment was more enlarged, likely including smaller airways. In contrast, using anatomic optical coherence tomography, in patients with asthma, COPD and bronchiectasis, AWC significantly decreased progressively as airway generation increased from 0 to 5, although specific AWC did not differ appreciably across airway generations [18].

If the measurements done at medium–low lung volumes are indeed reflective of the peripheral airways, then the mechanical properties measured by the technique we adopted in this study are clearly different than the mechanical ones measured by Williamson et al., who estimated compliances of the larger airways that they could access using the optical probe [18]. Therefore, the relationship between central airway compliance and that of more peripheral airways in patients with chronic airflow obstruction is unclear and needs further investigation.

After acute inhalation of bronchodilator, the airway wall became more compliant not only in healthy subjects but also in these COPD patients, potentially increasing the collapsibility of the airway and so limiting the maximal expiratory flow at each lung volume in the presence of high negative transmural pressure, as happens at different choke points along the intrathoracic airways during a forced expiratory maneuver. This contrasting effect induced by bronchodilating drugs should be considered when bronchial responsiveness is assessed by looking at the change of maximal flows and time-related volumes (i.e., FEV_1_) during a forced expiratory maneuver in patients with an obstructive ventilatory defect, like those who suffer from COPD. In fact, the possible bronchodilator-induced increase of small airway caliber due to greater cross-sectional internal surface area could be partly masked by increased airway wall compliance that becomes relevant during expiratory efforts in terms of final maximal expiratory flows obtained at any given lung volume.

Direct measurements before and after bronchodilator of either airway flow resistance by plethysmography at functional residual capacity or respiratory system flow resistance at different frequencies during tidal volume by forced oscillation technique (FOT) could easily avoid this problem, showing the real beneficial effect of bronchodilators, if any, in COPD patients.

The small number of patients is a limitation of this study because of possible type 1 error, as well as the inherent limitations of the method due to the impossibility of determining the exact location of the choke point that might be different between COPD patients and healthy subjects at relative iso-volumes. Finally, these findings cannot be generalized to all COPD patients, given the wide range of airflow severity and both physiologic and anatomic phenotypes in COPD.

## Conclusion

In conclusion, the findings of this study indicate that AWC measured at medium and low lung volumes, corresponding to the downstream segment of large and both large and small airways, respectively, is lower in COPD patients affected by chronic bronchiolitis than in matched control subjects, and notably, that bronchodilator does significantly increase AWC in COPD patients with mild-to-moderate airflow obstruction. It can be inferred that after bronchodilator, the collapsibility of the airway may be greater, due to AWC increase, under high positive pleural pressure, limiting the improvement of their caliber as assessed by the FEV_1_ changes during a forced expiratory maneuver and thus underestimating the effective bronchodilation achieved in the responsive COPD patients.
